# Clinical Profile, Renal Involvement, and Relapse Patterns in Pediatric Henoch–Schönlein Purpura: A Retrospective Observational Study from a Tertiary Care Centre in South India

**DOI:** 10.3390/children12101419

**Published:** 2025-10-21

**Authors:** Shrikiran A. Hebbar, Subramanyam Sheshadri, Praveen C. Samuel, Juanitha George, Suneel C. Mundkur, Pushpa Kini, Ramesh Bhat Y., Leslie Edward S. Lewis

**Affiliations:** Department of Pediatrics, Kasturba Medical College, Manipal, Manipal Academy of Higher Education, Manipal, Udupi 576104, Karnataka, India; shrikiran.a@manipal.edu (S.A.H.); subramanyam.sheshadri@nhs.net (S.S.); juanitha.george@manipal.edu (J.G.); suneel.mundkur@manipal.edu (S.C.M.); pushpa.kini@manipal.edu (P.K.); ramesh.bhaty@manipal.edu (R.B.Y.); leslie.lewis@manipal.edu (L.E.S.L.)

**Keywords:** Henoch–Schönlein purpura, IgA vasculitis, pediatric vasculitis, renal involvement, relapse, India, steroids, dapsone

## Abstract

**Highlights:**

**What are the main findings?**

• In this five-year, single-centre South-Indian pediatric cohort of Henoch–Schönlein purpura (HSP; n = 41), palpable purpura was universal, most often on the lower limbs (~95%), with frequent joint involvement (73.1%) and abdominal pain (61.0%) at presentation. Renal involvement occurred in 17% and was observed only in children aged ≥6 years, typically as microscopic haematuria with proteinuria (ISKDC II–III in the two biopsied cases). Rash relapse was uncommon (7.3%) and clustered with joint and abdominal symptoms at baseline; no relapsed child had nephritis, suggesting that the biology of cutaneous recurrence and renal morbidity may differ.
• Management reflected severity (NSAIDs 71.6%, corticosteroids 31.7%, dapsone 24.4%), and renal outcomes were favourable at a mean 18.9-month follow-up (one child on long-term antihypertensives; no progression to end-stage renal disease). Clinically, these data support age-targeted urine and BP surveillance from ≥6 years. It also indicates that relapse risk tracks with systemic (joint/abdominal) features rather than renal disease.

**What is the implication of the main finding?**

• Age ≥6 years should be treated as a pragmatic bedside flag for nephritis risk in HSP. Prioritize early and frequent surveillance (urine dipstick and blood pressure weekly for the first month, then monthly up to 6 months).
• Concurrent joint and abdominal symptoms at onset identify children at higher risk of cutaneous relapse rather than renal disease; plan relapse-focused follow-up and counselling accordingly.
• Routine inflammatory markers (ESR/CRP) were not predictive of nephritis and should not replace targeted clinical monitoring.

**Abstract:**

**Background/Objectives**: Henoch–Schönlein purpura (HSP), or IgA vasculitis, is the most common small-vessel vasculitis in children, yet Indian cohort data remain limited. We aimed to describe the clinical profile, renal involvement, treatment patterns, relapse, and outcomes of pediatric HSP at a tertiary centre in South India. **Methods:** We conducted a retrospective review of children <18 years diagnosed with HSP (January 2013–October 2018) using EULAR/PRINTO/PRES criteria. Demographics, clinical features, laboratory parameters, treatments, and outcomes were abstracted from records and analyzed in SPSS (descriptive statistics; Chi-square/Fisher’s exact and *t*/non-parametric tests as appropriate). Subgroup comparisons included renal vs. non-renal disease and age <6 vs. ≥6 years. An exploratory analysis examined predictors of nephritis. **Results:** Of 43 children identified, 2 were excluded (misclassified as systemic lupus erythematosus); 41 were analyzed. Mean age was 8.5 years (range 3–17), male: female 1.4:1. A preceding febrile illness or upper respiratory tract infection was noted in 41.4% and 17%, respectively. Palpable purpura was universal; joint involvement 73.1%, abdominal pain 61.0%, vomiting 41.5%. Renal involvement 17% occurred only in children ≥6 years; exploratory testing supported a strong age-linked signal for nephritis. Laboratory abnormalities included anemia (48.7%), thrombocytosis (19.5%), and elevated ESR (51.2%). Skin biopsy (n = 29) showed IgA and complement deposition; renal biopsy (n = 2) showed ISKDC grades II–III. Treatments included NSAIDs 71.6%, corticosteroids 31.7%, and dapsone 24.4% (used for severe systemic/persistent cutaneous disease). Rash relapse 7.3% clustered with joint plus abdominal symptoms and was not observed among children with nephritis. At a mean 18.9-month follow-up, one child required long-term antihypertensives; no child progressed to end-stage renal disease. **Conclusions:** Pediatric HSP in this South-Indian cohort followed a largely self-limited course with favourable renal outcomes. Age ≥6 years flagged higher renal risk, supporting age-targeted urine and blood-pressure surveillance, while relapse appeared to follow a non-renal trajectory (joint/abdominal clustering). Steroid and dapsone use reflected clinical severity rather than relapse risk. Findings align with Indian series and suggest lower renal morbidity than some East-Asian reports, adding region-specific evidence to guide monitoring and counselling.

## 1. Introduction

Henoch–Schönlein purpura (HSP), now termed Immunoglobulin A (IgA) vasculitis, is the most common systemic small-vessel vasculitis in children, characterized histologically by leukocytoclastic vasculitis with predominant IgA immune complex deposition. Clinically, it manifests with a tetrad of palpable purpura, arthralgia or arthritis, gastrointestinal involvement, and renal manifestations, with varying frequencies across regions and ethnic groups. The annual incidence among children is estimated at 10–20 per 100,000, with a male predilection and peak onset between 4 and 10 years of age [1,2,3]. Despite its usually self-limiting nature, renal involvement remains a key determinant of prognosis, occurring in approximately 20–50% of pediatric cases, potentially leading to chronic kidney disease in 5–10% [4,5,6,7]. Multiple studies have shown significant variation in the clinical presentation and disease course of HSP across populations. Indian studies by Mishra et al., Krishnan et al., and Sharma et al. have reported a predominance of abdominal pain and joint involvement, with renal disease rates ranging from 15 to 30% [2,4,8]. In particular, Peethambaran et al. highlighted the burden of renal involvement in South Indian children and the challenge of early prediction in low-resource settings [5].

On a global scale, Oni and Sampath have emphasized the pathophysiological complexity of HSP and current gaps in predictive biomarkers [9]. Trapani et al. and Breda et al. analyzed large European cohorts and confirmed the role of age and seasonality in disease expression and renal outcomes [6,10]. Epidemiological insights from Asia show a strong association with viral triggers and allergic diathesis, as seen in studies from Korea and China [3,7,11,12]. Villatoro-Villar et al. further established clinical differences between pediatric and adult HSP cases based on biopsy-confirmed diagnoses [13]. Although predictive models for nephritis risk based on age, gastrointestinal involvement, and laboratory parameters have been proposed in Western and East Asian literature [7,11], there is a dearth of such analysis from Indian cohorts. This creates an urgent need for locally relevant, evidence-based characterizations of disease profiles to inform clinical practice.

This retrospective observational study aims to delineate the clinical characteristics, laboratory findings, renal outcomes, treatment patterns, and relapse rates among children diagnosed with HSP at a tertiary care centre in South India. By comparing our data with national and international literature, we seek to enrich the understanding of disease variability, aid in early risk identification, and support context-specific treatment decisions for pediatric HSP in the Indian subcontinent.

## 2. Methods

### 2.1. Study Design and Setting

This retrospective observational study was conducted at the Department of Pediatrics, Kasturba Medical College Hospital, Manipal, a tertiary care academic centre in coastal South India. Medical records of children aged below 18 years, diagnosed with HSP between January 2013 and October 2018, were systematically reviewed. Follow-up data were extracted until July 2020. This study was conducted in accordance with the principles outlined in the Declaration of Helsinki. Ethical clearance was obtained from the Institutional Ethics Committee of Kasturba Medical College, Manipal Academy of Higher Education, Manipal (IEC:895/2018, 02/12/2018). All patient records were handled with strict confidentiality, and data were de-identified prior to analysis to ensure privacy and compliance with institutional data protection standards. Informed consent was obtained from all the participants of the study, and consent to publication of data was also obtained. Clinical trial registration was not required as this was a retrospective observational study.

### 2.2. Inclusion and Exclusion Criteria

**Figure 1:**
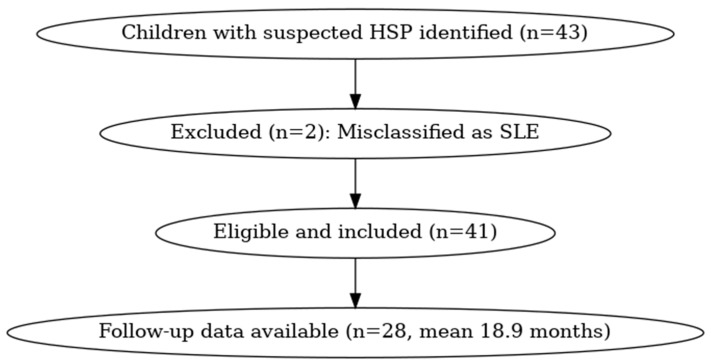
Flow diagram of patient inclusion and exclusion in the study cohort. HSP: Henoch–Schönlein Purpura, SLE: Systemic Lupus Erythematosus.

Children were included if they met the EULAR (European Alliance of Associations for Rheumatology)/PRINTO (Pediatric Rheumatology International Trials Organization)/PRES (Pediatric Rheumatology European Society) consensus classification criteria for HSP, which require the presence of palpable purpura with at least one of the following: arthritis or arthralgia, abdominal pain, or renal involvement. These diagnostic criteria have been validated in large multicentre studies and remain the standard framework globally [7,9,13]. Patients with alternative causes of purpura, such as thrombocytopenia, meningococcaemia, systemic lupus erythematosus, or drug-induced vasculitis, were excluded to avoid misclassification and ensure specificity (Figure 1) [1,6].

### 2.3. Operational Definitions and Treatment Modalities

Standardized operational definitions were employed based on prior studies to enable comparability. Palpable purpura was defined as non-blanching, raised purpuric lesions over dependent areas. Arthralgia or arthritis was recorded when joint pain, swelling, or restricted movement was noted. Renal involvement was defined by the presence of microscopic haematuria (≥5 RBCs/high power field), proteinuria (>0.3 g/day or spot urine protein: creatinine ratio >30 mg/g), or hypertension (blood pressure >95th percentile for age, sex, and height). These definitions were consistent with established Indian and international literature [4,8,11]. Treatment modalities recorded included non-steroidal anti-inflammatory drugs (NSAIDs), corticosteroids (oral or intravenous), dapsone, and antihypertensive agents. The rationale and indications for systemic therapy were documented when available. Outcome data included rash recurrence, persistence of renal involvement, and need for prolonged therapy.

### 2.4. Data Collection

Data were extracted using a structured template that included demographic characteristics, clinical manifestations, laboratory investigations, imaging findings, treatment administered, and follow-up outcomes. Clinical features assessed included rash distribution, joint involvement, gastrointestinal symptoms, and renal signs. Laboratory investigations included haemoglobin levels, total leukocyte count, platelet count, erythrocyte sedimentation rate (ESR), C-reactive protein (CRP), serum creatinine, urinalysis, urine protein-creatinine ratio, serum complement (C3), antinuclear antibody (ANA), and antistreptolysin O (ASO) titers. Imaging studies, when available, included abdominal ultrasonography to evaluate bowel wall thickening or intussusception. Skin biopsies were performed in a subset of patients and examined for features of leukocytoclastic vasculitis with direct immunofluorescence to detect IgA, C3, and fibrinogen deposition [5,13]. Renal biopsies were undertaken when clinically indicated and staged according to the International Study of Kidney Disease in Children (ISKDC) classification system [2,10].

### 2.5. Statistical Analysis

Data were analyzed using IBM SPSS Statistics version 26.0. Descriptive statistics were used to report frequencies and distributions. Chi-square test or Fisher’s exact test was used for categorical variables, while Student’s *t*-test or Mann–Whitney U test was applied for continuous variables, depending on the normality of distribution. Statistical methods were selected based on prior high-quality observational studies on HSP in pediatric cohorts across diverse populations [3,8,14].

## 3. Results

### 3.1. Demographic, Clinical, Laboratory and Treatment Profile

Out of 43 children initially identified, 2 were excluded due to a final diagnosis of systemic lupus erythematosus (SLE). The final study cohort comprised 41 children with Henoch-Schönlein purpura (HSP). The mean age at presentation was 8.5 years (range: 3–17 years), with a male-to-female ratio of 1.4:1. A preceding upper respiratory tract infection (URTI) was reported in 17%, and 41.4% presented with non-specific febrile illnesses. Among the 30 children with articular symptoms, the ankle (60.9%) and knee (46.3%) were the most frequently affected joints (Table 1). All patients received supportive care, while 71.6% were prescribed NSAIDs for symptom control. Corticosteroids were used in 31.7% of cases, typically for children with severe abdominal pain, joint symptoms, or renal involvement, with prednisolone initiated at a median dose of 1 mg/kg/day. Dapsone was administered in 24.4%, mainly for recurrent or steroid-refractory cutaneous manifestations. Two children (4.8%) required antihypertensives, and one remained on long-term therapy at five-year follow-up. Of the 41 children, 28 were followed for a mean duration of 18.9 months. There were no cases of end-stage renal disease, and arthritis did not relapse in any child.

### 3.2. Renal Manifestations

Among the seven children with renal involvement, microscopic haematuria with proteinuria was the most common presentation (85.7%), with a mean daily urinary protein excretion of 0.8 ± 0.3 g or a mean spot protein/creatinine ratio of 120 ± 45 mg/mmol. Two children (28.5%) presented with nephrotic syndrome, characterized by heavy proteinuria and hypoalbuminemia, while one child (14.3%) had acute nephritic syndrome with haematuria, hypertension, and transient elevation of serum creatinine. Hypertension was observed in two children, one of whom required long-term antihypertensive therapy at five-year follow-up. At the end of follow-up, mean serum creatinine remained within the normal pediatric range (0.6 ± 0.2 mg/dL), and no cases progressed to chronic kidney disease or end-stage renal disease. (Table 2).

### 3.3. Subgroup Analysis

Children with renal involvement (n = 7) were older (mean age 9.8 ± 2.1 years) compared to those without nephritis (8.2 ± 3.3 years), although the difference did not reach statistical significance (*p* = 0.19). Abdominal pain (85.7% vs. 56.0%) and vomiting (57.1% vs. 38.2%) were more frequent in the renal subgroup, but these associations were not statistically significant. Importantly, the use of systemic corticosteroids (85.7% vs. 17.6%, *p* < 0.001) and dapsone (57.1% vs. 17.6%, *p* = 0.02) was significantly higher among children with renal disease, reflecting greater clinical severity. Rash relapse was not observed in the renal subgroup, whereas it occurred in 8.8% of those without nephritis (*p* = 0.40). When stratified by age, none of the children younger than 6 years developed nephritis, whereas 24.1% of those aged ≥ 6 years were affected (*p* < 0.001). Older children were also more likely to receive corticosteroids (37.9% vs. 8.3%, *p* = 0.04) and dapsone (31.0% vs. 8.3%, *p* = 0.07). Rash relapse was observed exclusively in the ≥6 year group (10.3%), though the association was not statistically significant. These findings suggest that age above 6 years is a strong predictor of nephritis, while systemic involvement influences therapeutic escalation (Table 3). In post hoc analyses (Table 4), age ≥ 6 years showed the largest association with nephritis (OR 8.33, 95% CI 0.44–158.43; Fisher *p* = 0.085), consistent with absence of events in <6 years and small-sample constraints. Abdominal pain and vomiting were not associated with nephritis (*p* > 0.20). Steroid (OR 19.00, 95% CI 2.65–136.13; *p* = 0.001) and dapsone use (OR 5.64, 95% CI 1.10–28.95; *p* = 0.047) were higher among children with nephritis, reflecting treatment escalation for clinical severity rather than baseline risk. Clinical implication: prioritize urine and blood-pressure monitoring in children ≥6 years (Table 4).

### 3.4. Predictors of Rash Relapse

Rash relapse occurred in three children (7.3%), all of whom were aged ≥6 years. Every relapsed case presented with both joint involvement and abdominal pain at baseline, whereas none had renal disease. Compared with the non-relapse group, joint and abdominal symptoms were more frequent, although differences did not reach statistical significance (joint involvement 100% vs. 71.1%, *p* = 0.56; abdominal pain 100% vs. 57.9%, *p* = 0.28). Notably, none of the relapsed patients had nephritis, suggesting that relapse and renal morbidity follow distinct pathways. The use of corticosteroids (33.3% vs. 31.6%, *p* = 0.99) and dapsone (33.3% vs. 21.1%, *p* = 0.56) did not differ between groups, indicating that therapeutic interventions were unlikely to explain recurrence patterns (Table 5).

## 4. Discussion

Henoch–Schönlein purpura (HSP), or IgA vasculitis, remains the most common systemic small-vessel vasculitis in childhood. In our single-centre South Indian cohort (n = 41), we observed a characteristic clinical spectrum with universal palpable purpura, high rates of joint and gastrointestinal symptoms, and a 17% prevalence of nephritis, all in children aged ≥ 6 years. Rash relapse was uncommon (7.3%) and clustered with joint plus abdominal involvement rather than renal disease. Supportive care was universal, NSAIDs were used in 71.6%, corticosteroids in 31.7%, and dapsone in 24.4%, with reassuring renal outcomes at follow-up (mean 18.9 months). These observations align with both Indian and international literature while providing region-specific insights into age-linked renal risk and relapse patterns. Our cohort’s renal involvement (17%) falls squarely within the 5–22% range typically reported in Indian series [2,4,5,8] and 11–18% in Chinese cohorts [7,11], but is less than 28–54% reported by European studies [6,10]. The renal phenotype was predominantly microscopic haematuria with proteinuria, with only infrequent nephrotic/nephritic syndromes or hypertension, consistent with a comparatively milder renal course in our setting. Table 6 summarizes these cross-cohort contrasts and situates our data alongside Indian, Chinese, and European series [6,10].

A central finding is the age-linked gradient in renal risk: no child <6 years developed nephritis, whereas 24.1% of those ≥6 years were affected. This practical bedside signal complements prior large cohorts that have implicated older age in renal morbidity and, in our series, reached statistical significance (*p* = 0.01) on subgroup testing. Clinically, this supports targeted urine surveillance and blood pressure monitoring in school-age children with HSP, especially within the first month when renal findings most often emerge.

Although relapse was infrequent, every relapsed child presented with joint and abdominal symptoms at baseline, and none had nephritis, suggesting that cutaneous recurrence and renal disease may follow separable trajectories. Therapeutic exposure (steroids/dapsone) did not differ meaningfully between relapsed and non-relapsed groups, indicating that relapse in our cohort likely reflects disease biology more than treatment decisions. This pattern echoes prior observations that systemic (non-renal) features can track with rash recurrence [9,13].

Corticosteroids were used in roughly one-third of children—most often for severe abdominal or joint symptoms—and were understandably more common among those with renal involvement and in older children (≥6 years), mirroring higher systemic burden. Dapsone (24.4%) was employed as an adjunct for relapsing or steroid-refractory rash, an approach infrequently reported in the literature and deserving of prospective evaluation. Importantly, we observed no progression to end-stage renal disease during follow-up, and only one child required long-term antihypertensive therapy—a reassuring medium-term renal outlook that nonetheless underscores the need for continued surveillance beyond the cutaneous phase [13,14]. Case ascertainment used EULAR/PRINTO/PRES classification; renal biopsies (when indicated) were staged per ISKDC—approaches in line with prior high-quality pediatric cohorts and ensuring comparability with global literature [2,9,10,11,14]. Together with skin-biopsy immunopathology in most tested children, this underpins diagnostic rigour and cohort fidelity.

Strengths include a well-characterized regional cohort, explicit age-stratified analyses, and a consolidated presentation of mean ± SD laboratory values, *p*-values for subgroup comparisons, and quantitative urinary protein data—addressing gaps noted in prior pediatric series. Limitations reflect the retrospective, single-centre design and modest sample size, which restrict power for multivariable modelling and may introduce selection and documentation biases. For frontline care, three pragmatic points emerge: (1) age ≥6 years is a useful bedside flag for renal surveillance; (2) relapse risk clusters with joint + abdominal symptoms rather than renal disease, guiding anticipatory counselling and follow-up; and (3) most children follow a self-limited course with favourable renal outcomes, provided monitoring is systematic through and beyond the initial month. These insights, aligned with national and international experience, can help standardize care pathways in similar resource settings [3,4,5,6,8,9,10,11,13,14].

## 5. Conclusions

Henoch–Schönlein purpura (IgA vasculitis) in children is a largely self-limiting condition with favourable outcomes in most cases, though renal involvement remains the key determinant of long-term prognosis. Across studies, renal morbidity shows considerable geographic and ethnic variation, while relapse patterns appear more closely linked to systemic symptoms such as joint and abdominal involvement rather than kidney disease. Age consistently emerges as an important risk factor, with older children showing a greater likelihood of nephritis, underscoring the need for targeted urine and blood pressure monitoring. Overall, timely recognition, age-appropriate surveillance, and judicious use of corticosteroids or adjunctive agents can optimize outcomes and reduce the burden of chronic complications.

## Figures and Tables

**Table 1:** Baseline demographic, clinical, and laboratory characteristics of children with Henoch–Schönlein purpura (n = 41).

Parameter	Value
**Demographics**	
Mean age, years (range)	8.5 ± 3.2 (3–17)
Male:Female ratio	1.4:1
Preceding febrile illness	41.4%
Preceding upper respiratory tract infection	17%
**Clinical presentation**	
Palpable purpura	100%
Joint involvement	73.1%
Abdominal pain	61%
Vomiting	41.5%
Gastrointestinal bleeding	9.7%
Renal involvement	17%
Rash relapse	7.3%
**Laboratory findings**	
Haemoglobin (g/dL)	11.8 ± 1.9 (Anemia: 48.7%)
WBC count (/mm^3^)	12,000 [10,500–15,000]
Platelet count (/mm^3^)	350,000 [280,000–420,000]; Thrombocytosis: 19.5%
ESR (mm/hr)	35 ± 12 (elevated in 51.2%)
CRP (mg/L) *	12.5 ± 6.2
ASO titre (IU/mL) *	180 ± 65
Serum creatinine (mg/dL)	0.6 ± 0.2 (all within reference range)
C3 complement	Normal in all tested
**Joint Affected**	
Ankle	60.9%
Knee	46.3%
Hip	5.1%
Elbow	17%
Wrist	12.2%
Multi-joint involvement	46.3%
**Imaging/biopsy**	
Abdominal USG abnormality	56.6% (bowel wall changes)
Skin biopsy (n = 29)	Leukocytoclastic vasculitis with IgA, C3, fibrinogen
Renal biopsy (n = 2)	ISKDC Grade II and III

Values are expressed as mean ± standard deviation, median [IQR], or percentage. * CRP and ASO values were available for a subset of patients. ESR cutoff for elevation defined as >20 mm/hr. Anemia defined as Hb < 11 g/dL; thrombocytosis as platelet count > 450,000/mm^3^. USG = ultrasonography; ASO = antistreptolysin O; CRP = C-reactive protein; ESR = erythrocyte sedimentation rate; C3 = complement component 3; ISKDC = International Study of Kidney Disease in Children.

**Table 2:** Renal manifestations in children with HSP nephritis (n = 7).

Renal Finding	Frequency (%)	Quantitative Data
Microscopic haematuria with proteinuria	85.7	Mean urine protein: 0.8 ± 0.3 g/day (spot PCR 120 ± 45 mg/mmol), urine RBC > 5 RBCs/hpf
Nephrotic syndrome	28.5	Proteinuria > 40 mg/m^2^/hr; serum albumin < 2.5 g/dL
Acute nephritic syndrome	14.3	Haematuria with hypertension; serum creatinine 0.9 ± 0.2 mg/dL
Hypertension	28.6 (2/7)	One child required long-term antihypertensives
Normal renal function (at last follow-up)	100 (7/7)	Mean serum creatinine 0.6 ± 0.2 mg/dL

Percentages are calculated within the nephritis subgroup (n = 7). Urinary protein levels expressed as mean ± SD; spot PCR = urine protein/creatinine ratio. Hypertension is defined as BP >95th percentile for age/sex/height. Nephrotic syndrome is defined as proteinuria >40 mg/m^2^/hr plus hypoalbuminemia (<2.5 g/dL). Acute nephritic syndrome is defined as haematuria with hypertension and variable renal impairment.

**Table 3:** Subgroup analysis of clinical features and treatments in children with HSP (n = 41).

Parameter	Renal (n = 7)	Non-Renal (n = 34)	*p*-Value	Age < 6 Years (n = 12)	Age ≥ 6 Years (n = 29)	*p*-Value
Mean age (years)	9.8 ± 2.1	8.2 ± 3.3	0.19	4.5 ± 1.0	10.0 ± 2.6	<0.001 ***
Abdominal pain (%)	85.7	56.0	0.12	50.0	65.5	0.33
Vomiting (%)	57.1	38.2	0.41	33.3	44.8	0.51
Steroid use (%)	85.7	17.6	<0.001	8.3	37.9	0.04 *
Dapsone use (%)	57.1	17.6	0.02	8.3	31.0	0.07
Rash relapse (%)	0	8.8	0.40	0	10.3	0.28

Values are presented as mean ± standard deviation (SD) or percentage. *p*-values were derived using Student’s *t*-test for continuous variables (age) and Chi-square or Fisher’s exact test for categorical variables. “Renal involvement” was defined by the presence of haematuria and/or proteinuria with or without hypertension. “Rash relapse” was defined as recurrence of cutaneous lesions ≥ 2 weeks after initial resolution. Dapsone dose: median 2 mg/kg/day; Prednisolone dose: median 1 mg/kg/day. *** *p* value very high significance (<0.001), * *p* value significant (<0.05).

**Table 4:** Predictors/associations with nephritis (renal involvement)—exploratory analyses.

Predictor	Definition (Coding)	OR (Haldane)	95% CI	*p* (Fisher, 2-Sided)
Age ≥ 6 years	≥6 y = 1; <6 y = 0 (7/29 vs. 0/12)	8.33	0.44–158.43	0.085
Abdominal pain	Yes vs. No (6/7 vs. 19/34)	3.44	0.52–22.95	0.215
Vomiting	Yes vs. No (4/7 vs. 13/34)	2.05	0.43–9.68	0.421
Steroid use ^†^	Any steroid vs. none (6/7 vs. 6/34)	19.00	2.65–136.13	0.001
Dapsone use ^†^	Any dapsone vs. none (4/7 vs. 6/34)	5.64	1.10–28.95	0.047

ORs computed with Haldane–Anscombe correction; CIs by log-Wald approximation; *p* values by two-sided Fisher’s exact. Counts shown in parentheses are “event/total” in the renal group vs. the non-renal group used for the 2 × 2 tests. ^†^ Treatment rows reflect association with clinical severity, not baseline predictors; interpret as management intensity rather than causal risk factors. (Dosing ranges—prednisolone ~ 1 mg/kg/day; dapsone ~ 2 mg/kg/day).

**Table 5:** Predictors of rash relapse (n = 3 relapsed cases).

Predictor	Relapse Group (n = 3)	Non-Relapse Group (n = 38)	*p*-Value
Joint involvement	100%	71.1%	0.56
Abdominal pain	100%	57.9%	0.28
Renal involvement	0%	18.4%	0.99
Steroid use	33.3%	31.6%	0.99
Dapsone use	33.3%	21.1%	0.56

Values presented as percentages within each subgroup. *p*-values calculated using Fisher’s exact test. “Relapse” defined as recurrence of rash ≥ 2 weeks after resolution. No patient with renal involvement experienced relapse.

**Table 6:** Comparative summary of pediatric HSP cohorts (our study vs. prior literature).

Study (Region)	Period/N	Renal Involvement (%)	Rash Relapse (%)	Steroid Use (%)	Dapsone Use (%)	Key Notes
Present study—South India	2013–2018/41	17	7.3	31.7	24.4	Nephritis only in ≥6 y; phenotype predominantly microscopic haematuria + proteinuria
Mishra (India, 2019) [2]	2016–2018/27	22	NS	94.4	NS	Indian series; renal disease within 15–30% range
Krishnan (India, 2018) [4]	2015–2017/61	5	NS	55	NS	Reports frequent joint/abdominal involvement
Sharma (India, 2022) [8]	2021/65	5	NS	NS	NS	Similar renal burden; relapse data not available
Peethambaran (South India) [5]	2012–2014/64	23.5	23.5	23	NS	Higher renal burden; early prediction is challenging in low-resource settings
Xu (China, 2019) [11]	2015–2017/1200	18.1	10.2	NS	NS	Higher renal morbidity than our cohort
Chen (China, 2016) [7]	2000–2008/2240	11	NS	NS	NS	Higher renal morbidity than our cohort
Trapani (Europe, 2005) [6]	1998–2002/150	54	NS	NS	NS	Seasonal- and age-related risk signals
Breda (Europe, 2021) [10]	2000–2016/208	28	NS	30	NS	Renal risk varies regionally; multicentre cohort

NS = Not specified; y = years.

## Data Availability

The raw data supporting the conclusions of this article will be made available by the authors on request.

## Abbreviations

ANA	Antinuclear Antibody
ASO	Antistreptolysin O
CKD	Chronic Kidney Disease
CRP	C-Reactive Protein
ESR	Erythrocyte Sedimentation Rate
EULAR	European Alliance of Associations for Rheumatology
GI	Gastrointestinal
HSP	Henoch–Schönlein Purpura
IgA	Immunoglobulin A
ISKDC	International Study of Kidney Disease in Children
NSAIDs	Non-Steroidal Anti-Inflammatory Drugs
PRES	Pediatric Rheumatology European Society
PRINTO	Pediatric Rheumatology International Trials Organization
SLE	Systemic Lupus Erythematosus
SPSS	Statistical Package for the Social Sciences
URTI	Upper Respiratory Tract Infection
USG	Ultrasonography
